# Intravenous thrombolysis in combination with mild hypothermia therapy in the treatment of acute cerebral infarction

**DOI:** 10.12669/pjms.35.4.311

**Published:** 2019

**Authors:** Xiaoying Liu, Shengli Rao, Jiajia Wang

**Affiliations:** 1 Xiaoying Liu Departments of Neurology, Binzhou People’s Hospital, Shandong, 256610, China; 2 Shengli Rao Departments of Emergency, Binzhou People’s Hospital, Shandong, 256610, China; 3 Jiajia Wang Departments of Neurology, Binzhou People’s Hospital, Shandong, 256610, China

**Keywords:** Acute cerebral infarction, Intravenous thrombolysis, Mild hypothermia therapy

## Abstract

**Objective:**

To investigate the efficacy of recombinant tissue plasminogen activator (rt-PA) intravenous thrombolysis in combination with mild hypothermia therapy in the treatment of acute cerebral infarction.

**Methods:**

One hundred and thirty-two patients with acute cerebral infarction who were admitted to our hospital were selected and grouped into a control group and an observation group, 66 each group. Patients in the control group were given conventional treatment in combination with local mild hypothermia therapy, and patients in the observation group were given rt-PA intravenous thrombolysis on the basis of conventional treatment and local mild hypothermia therapy. National institute of health stroke scale (NIHSS) score and intracranial pressure (ICP) of the two groups before and after treatment was recorded. The efficacy of the two groups was evaluated. The modified Rankin scale (MRS) score was followed up for three months. The blood samples of the patients were collected before and after thrombolysis. Superoxide dismutase (SOD) and malondialdehyde (MDA) levels in the plasma were detected.

**Results:**

The NIHSS score of the two groups decreased in the 1^st^, 3^rd^ and 7^th^ day after treatment compared to before treatment (p<0.05), but the NIHSS score of the two groups had no significant difference at different time points after treatment (p>0.05). The ICP of the two groups decreased in the 1^st^, 3^rd^ and 7^th^ day after treatment compared to before treatment (p<0.05), and the decrease of ICP of the observation group was more significant than that of the control group at the same time point (1^st^, 3^rd^ and 7^th^ day after treatment) (p<0.05). The clinical efficacy of the observation group was higher than that of the control group after treatment, and the difference was statistically significant (p<0.05). The MDA concentration of both groups decreased at different time points after treatment (p<0.05), but the SOD concentration increased (p<0.05). The MDA concentration of the observation group was lower than that of the control group at different time points after treatment (p<0.05), and the SOD concentration of the observation group was higher than that of the control group (p<0.05).

**Conclusion:**

rt-PA intravenous thrombolysis in combination with mild hypothermia therapy has significant efficacy in the treatment of acute cerebral infarction. It can effectively relieve neurological function. Its action mechanism may be realized by relieving oxidative stress response.

## INTRODUCTION

Acute cerebral infarction, also known as acute ischemic stroke, refers to neurological deficit syndrome caused by necrosis and softening of brain tissue caused by ischemia and hypoxia, and its incidence was 60%~70% among all stroke.^1^,^2^ With the aggravation of population aging in China, the incidence of acute cerebral infarction has increased year by year. Its high mortality and disability rate have seriously affected the health and quality of life of patients.^3^ Relevant studies have shown that oxidative stress is one of the basic mechanisms of nerve cell damage in stroke.^4^,^5^ Oxidative stress refers to tissue damage induced by the production of reactive oxygen species (ROS) in vivo and the imbalance between antioxidant defense systems. ROS can oxidize unsaturated fatty acid on cytomembrane, and the final product of the reaction is malondialdehyde (MDA). Superoxide dismutase (SOD) is an important antioxidant enzyme in organisms. The determination of MDA and SOD can indirectly reflect the oxidative and antioxidant capacity of organism.^6^ Clinical principles of acute cerebral infarction are to recanalize occluded blood vessels as soon as possible, improve blood perfusion in ischemic areas, and avoid neurological deficits. Therapeutic methods of acute cerebral infarction include thrombolysis, anticoagulation, anti-platelet aggregation, etc.^7^,^8^ Recombinant tissue plasminogen activator (rt-PA) is the only drug approved by the Food and Drug Administration (FDA) for intravenous thrombolysis of acute cerebral infarction.^9^ The thrombolytic window of rt-PA is within 4.5 hours after onset, especially within three hours after onset. rt-PA has been recommended as class-I in many national guidelines for the diagnosis and treatment of acute stroke because of its class A evidence-based medical evidence for thrombolytic therapy.^10^

According to the relevant literature,^11^ mild hypothermia treatment has a definite protective effect on patients with acute cerebral infarction, which is conducive to restoring and improving the neurological function of patients damaged in the acute phase of cerebral infarction. However, most scholars in China and abroad are devoted to the study of efficacy of single application of rt-PA intravenous thrombolysis and mild hypothermia treatment, but rarely reported the effect of rt-PA intravenous thrombolysis in combination with mild hypothermia treatment. Therefore, rt-PA intravenous thrombolysis was combined with local mild hypothermia therapy in this study, the therapeutic effect of the regimen in the treatment of acute cerebral infarction was observed, its impact on oxidative stress injury was detected, and the possible mechanism of action was analyzed. This work aims to guide the clinical treatment of acute cerebral infarction.

## METHODS

One hundred and thirty-two patients with acute cerebral infarction who were admitted to our hospital from August 2016 to August 2017 were selected and divided into an observation group and a control group according to random number table. There were 66 patients in each group. There were 32 males and 34 females in the control group, and they aged 46-82 years (average (62.13±9.36) years). The time from onset to treatment was 1-6 hours (average (3.13±0.56) h). As to complications, there were 35 cases of hypertension, 9 cases of atrial fibrillation and 22 cases of diabetes. The observation group consisted of 28 males and 38 females, and they aged 43-84 years (average (64.26±10.46) years). The time from onset to treatment was 1-5 hours (average (3.08±0.72) hours). As to complications, there were 36 cases of hypertension, nine cases of atrial fibrillation and 21 cases of diabetes. There was no significant difference in baseline data such as gender and age between the two groups. The content of this study has been approved by the medical ethics committee of our hospital, and all the subjects signed the informed consent voluntarily.

### Inclusive and exclusive criteria

Inclusive criteria included conforming to diagnostic criteria of acute cerebral infarction described in the Guidelines for the Diagnosis and Treatment of Acute Ischemic Stroke in China (2014) formulated by the Neurological Society of Chinese Medical Association,^12^ being found having new acute cerebral infarction by brain Magnetic Resonance Imaging (MRI) and diffusion weighted imaging (DWI), and duration of the first onset shorter than 4.5 hours. Exclusive criteria included having severe heart, liver, lung, kidney dysfunction and advanced malignant tumors, having other intracranial lesions or cerebral hemorrhage, platelet count < 100×10^9^/L in patients with abnormal coagulation function or undergoing anticoagulation therapy, undergoing major surgery or having a history of cerebrovascular accident in the past six months, and being suspected having posterior circulation infarction because of the existence of severe consciousness disorder or with NIHSS score higher than 25 points.

### Treatment methods

The control group was treated with conventional therapy and mild hypothermia. SDL-V double-controlled brain cooling instrument (Tangshan Northern Medical Equipment Co., Ltd., China) and Omron infrared ear chamber thermometer (Dalian Omron Co., Ltd., China) were used. The operation was as follows. The cooling pillow cushion was placed inside the helmet, and the bushing and helmet were put on to ensure that the medical cryotherapy instrument and helmet were at the same horizontal level. The temperature of ice cap was about 0°C, the brain temperature was about 34°C, and the temperature was automatically adjusted. The duration of mild hypothermia was 72 hours, and the blood potassium, electrocardiograph, pupil, blood pressure, pulse and breathing were detected. Temperature was improved slowly, 1°C/d, and the rewarming speed was lower than 0.1°C/h. On the basis of the treatment of the control group, the observation group was given rt-PA (Boehringer-Ingelheim Pharm, Germany; registration number: S20110052) intravenous thrombolysis, at a dose of 0.6 mg/kg. Firstly 10%-15% of the total dose of intravenous injection was intravenously injected within one minute, and the remaining dose was dissolved in 100 mL of normal saline and dripped through vein for one hour.

### Observational indicators

(*1) Neurological function score and ICP monitoring:* The NIHSS score and intracranial pressure of patients in the two groups were recorded before treatment and in the 1^st^, 3^rd^ and 7^th^ day after treatment. ICP was non-invasively detected by flash-visual evoked potential.^11^ Non-invasive ICP value was automatically calculated by corresponding program.

(2) *Evaluation of efficacy:* The efficacy was evaluated according to the criteria for NIHSS score of stroke approved by the the Fourth National Academic Conference on the Diagnosis of Cerebrovascular Disease (1995) in the 7^th^ day after treatment.^12^ Decrease of NIHSS score for 91%~100% was evaluated as basically cured; decrease for 46%~90% was evaluated as significantly progressive; decrease for 18%~45% was evaluated as effective; decrease for lower than 17% was evaluated as ineffective. The formula for the overall effective rate was: the overall effective rate=(number of basically cured cases+number of significantly progressively cases+number of effective cases)/total number of cases×100%.

*(3) Detection of SOD and MDA:* The peripheral venous blood of the two groups was collected 1, 3 and 7 days after treatment, centrifuged for 5 minutes at 3500 r/min, and stored at - 80°C. All indicators were determined by numbered double-blind method. SOD was determined by chemical colorimetry and MDA by thiobarbituric acid colorimetry. Kit (Nanjing Jiancheng Bioengineering Research Institute Co., Ltd., China) was used. Different kinds of reagents were prepared according to the kit instructions, and the preparation and determination steps strictly followed the operating procedures. A Shanghai 7200 spectrophotometer with 550 nm and 0.5 cm optical path was used.

### Statistical method

SPSS ver. 21.0 was used. Measurement data were expressed as Mean±SD and processed by analysis of variance. Enumeration data were expressed as percentage (%) and processed by Chi-square test. The significant level was α=0.05. Difference was considered as statistically significant if p<0.05.

## RESULTS

There was no significant difference in the NIHSS score between the two groups before treatment (p>0.05). The NIHSS score in the 1^st^, 3^rd^ and 7^th^ day after treatment was lower than that before treatment (p<0.05). There was no significant difference in the NIHSS score between the two groups in the 1^st^, 3^rd^ and 7^th^ day after treatment (p>0.05, Table-I).Similarly there was no significant difference in ICP between the two groups before treatment (p>0.05). ICP decreased in the 1^st^, 3^rd^ and 7^th^ day after treatment (p<0.05). ICP decreased more significantly in the observation group than in the control group at the same time point after treatment (p<0.05, Table-II).The overall efficacy of the observation group was 87.88% (58/66), which was significantly higher than that of the control group, 71.21% (47/66). The difference between the two groups had statistical significance (p<0.05, Table-III).

**Table I:** Comparison of NIHSS score between the two groups before and after treatment.

Group	NIHSS score			

	Before treatment	1 d after treatment	3 d after treatment	7 days after treatment
Observation group	15.68±6.39	11.77±5.92*	8.87±5.80*	5.45±3.41*
Control group	15.88±7.09	11.81±6.09*	9.37±6.35*	5.98±3.42*

Note:

* indicated p<0.05 compared to before treatment.

**Table II:** Comparison of ICP between the two groups before and after treatment.

Group	ICP			

	Before treatment	1 d after treatment	3 d after treatment	7 days after treatment
Observation group	237.12±65.58	163.45±42.36*^#^	143.66±34.54*^#^	132.45±24.07*^#^
Control group	237.65±68.24	183.52±51.54*	164.36±40.16*	149.47±24.09*

Note:

* indicated p<0.05 compared to before treatment;

# indicated p<0.05 compared to the control group.

**Table III:** Comparison of clinical efficacy between the two groups [n(%)].

Group	Basically cured	Significantly progressive	Effective	Ineffective	Overall effective rate
Observation group	24(36.36)	20(30.30)	14(21.21)	8(12.12)	58(87.88)
Control group	16(24.24)	14(21.21)	17(25.76)	19(28.79)	47(71.21)
X^2^					16.957
t					<0.05

Before treatment, there was no significant difference in the levels of SOD and MDA between the two groups (p>0.05); the level of serum SOD in the observation group in the 1^st^, 3^rd^ and 7^th^ day after treatment was higher than that before treatment, and the level of serum MDA was lower than that before treatment (p<0.05); the level of serum SOD in the control group in the 3^rd^ and 7^th^ day after treatment was higher than that before treatment, and the level of serum MDA was lower than that before treatment. (p<0.05). The serum SOD level of the observation group was significantly higher than that of the control group at the same time point after treatment, and the serum MDA level was significantly lower than that of the control group at the same time point after treatment (p<0.05, Table-IV).

**Table IV:** Comparison of the serum SOD and MDA levels between the two groups before and after treatment (mmol/L).

Group	Indicator	Before treatment	1 day after treatment	3 days after treatment	7 days after treatment
Observation group	SOD	72.42±17.28	85.63±12.54*^#^	97.35±9.88*^#^	110.59±10.00*^#^
	MDA	10.80±0.51	8.34±0.69*^#^	5.41±0.28*^#^	5.64±0.22*^#^
Control group	SOD	73.32±18.46	74.52±19.49	86.26±18.58*	92.53±19.46*
	MDA	10.88±0.92	10.46±0.88	7.13±0.42*	7.14±0.66*

Note:

* indicated p<0.05 compared to before treatment;

# indicated p<0.05 compared to the control group.

## DISCUSSION

Acute cerebral infarction is a common cerebrovascular disease. Its clinical symptoms are closely related to the location of lesion, the size and ischemia degree of cerebral ischemic vessels, and whether or not it is accompanied by important organ diseases. In mild cases, there are no symptoms, but dizziness and limb paralysis may occur repeatedly; in severe cases, coma or even death may occur, which is a severe threat to the life safety of patients.^13^ At present, the treatment of cerebral infarction has been the focus, hotspot and difficulty of clinical research.

Ultra-early intravenous thrombolytic therapy for acute cerebral infarction is considered to be one of the most effective therapies at present.^14^ In particular; rt-PA intravenous thrombolytic therapy for cerebral infarction has become the preferred choice recommended by national guidelines. rt-PA is a serine protease which can specifically bind fibrin to lyse the provaline-arginine junction, thus activating plasminogen, forming fibrinolytic enzyme, dissolving blood clots, promoting blood flow recovery in ischemic penumbra, saving nerve cells and promoting the recovery of nerve function in patients.^15^ In recent years, mild hypothermia has been recognized as an important method of neuroprotection. Mild hypothermia can improve the function of cerebral cortex, inhibit brain oxygen metabolism, protect blood-brain barrier, inhibit excessive release of neurotransmitters, inflammatory reaction and calcium overload, accelerate the growth of nerve cell trunk, and inhibit neuronal apoptosis, thereby alleviating and improving neurologic impairment.^16^ Su et al. also found that systemic mild hypothermia intervention could improve the clinical prognosis of massive cerebral infarction.^17^ However, the efficacy of mild hypothermia intervention in combination with intravenous thrombolysis has not been evaluated in China. Webster et al. found that rt-PA activity decreased by 2%-4% when body temperature dropped to 30-33°C.^18^ Therefore, it was assumed that intravenous thrombolysis combined with mild hypothermia was more effective than single therapy in treating acute cerebral infarction. The results of this study showed that the total clinical effective rate of the observation group was significantly higher than that of the control group (p<0.05). This result strongly supports that rt-PA intravenous thrombolysis combined with mild hypothermia can further improve the clinical effect in the treatment of acute cerebral infarction.

Moreover this study found that ICP of the two groups after 1^st^, 3^rd^ and 7^th^ days of treatment was lower than that before thrombolysis. ICP of the observation group was significantly lower than that of the control group after 3^rd^ and 7^th^ days of treatment. The reason might be that mild hypothermia relieved inflammatory reaction and brain cell edema to reduce ICP after cerebral infarction, and rt-PA intravenous thrombolysis recanalized blood vessels to achieve ischemic penumbra re-moistening, which significantly reduced ICP. Therefore, intravenous application of rt-PA combined with mild hypothermia had a high feasibility in the treatment of acute cerebral infarction and was of great significance to improve quality of life and reduce disability rate.

A large number of free radicals will be produced when neurocyte injury happens after cerebral infarction, which can further aggravate brain injury.^19^ Studies have shown that oxidative stress in neurons leads to death after cerebral ischemia and pathological processes such as increased blood-brain barrier permeability and brain edema are also closely related to it.^20^,^21^ Pluta et al. took the rat model of cerebral infarction as the research subject and found that rt-PA intravenous thrombolysis could relieve ischemia-reperfusion injury and reduce infarct size by alleviating oxidative reaction.^22^ Moreover a large number of studies have found that mild hypothermia therapy could effectively protect ischemic and traumatic brain injury and early implementation of mild hypothermia therapy in the early stage of cerebral ischemia-reperfusion could effectively avoid apoptosis.^23^,^24^ It can be seen that rt-PA intravenous thrombolysis and mild hypothermia can reduce oxidative stress after cerebral infarction. The results of this study showed that MDA concentration in the observation group was lower than that in the control group at the 1^st^, 3^rd^ and 7^th^ day after treatment, and SOD concentration was higher, suggesting that rt-PA intravenous thrombolysis combined with mild hypothermia could alleviate oxidative stress better, and it is the possible mechanism of reducing disability rate and gaining more long-term benefits.

## CONCLUSION

Intravenous thrombolysis combined with mild hypothermia on acute cerebral infarction is effective in improving the neurological function reducing oxidative stress response. However, this study still has the following shortcomings. The sample size was not large enough, which limited the scientificity of the experimental results, and randomized controlled study with a large sample size is needed to further verify the conclusion. Moreover complications of patients in the study process were not taken into account, and the safety of combined treatment needs further investigation.

### Authors’ Contribution

**XYL:** Study design, data collection and analysis.

**XYL & SLR:** Manuscript preparation, drafting and revising.

**XYL & JJW:** Review and final approval of manuscript.
